# Visual functions and disability in Iranian adults: a population-based study

**DOI:** 10.1186/s12886-022-02262-9

**Published:** 2022-01-20

**Authors:** Hassan Hashemi, Fatemeh Mehravar, Soheila Asgari, Mohammad Hassan Emamian, Akbar Fotouhi

**Affiliations:** 1 Noor Ophthalmology Research Center, Noor Eye Hospital, Tehran, Iran; 2 Department of Epidemiology and Biostatistics, School of Public Health, Tehran University of Medical Sciences, Tehran, Iran; 3 Ophthalmic Epidemiology Research Center, Shahroud University of Medical Sciences, Shahroud, Iran

**Keywords:** Visual function, Short-form visual functioning scale, NEI-VFQ, Population-based study

## Abstract

**Background:**

Vision-related quality of life is related to severity of visual impairments and show the impact of eye diseases on daily activities. This study aims to assess visual functions and disability and its association with age, gender, education, marital status, and economic status in adults aged 45–69 years.

**Methods:**

Data in this population-based study were from the second phase of the Shahroud eye cohort study and collected by using a Short-Form Visual Functioning Scale. The scores of visual function and disability were calculated based on Rasch-transformed scores of the National Eye Institute visual functioning questionnaire, where a more negative score indicates a better situation. Multiple linear regression was used to investigate the factors associated with visual functions.

**Results:**

Among 4737 participants the visual function data for 4715 people were analyzed. The visual function of 75.3, 17.1 and 7.5% of participants were “ideal and good”, “moderate”, and “bad and very bad”, respectively, while 0.06% were unable for vision. The running mean of the visual function was calculated to be − 3.95 ± 0.02. The visual performance was worse in females than the males (β = 0.14, *p* = 0.005). Visual function improved with increasing levels of education (β = − 1.06, *p* < 0.001). It was worse in low-economic (β = 0.016, p = 0.005) and moderate-economic (β = 0.28, p < 0.001) participants than high-economic ones.

**Conclusion:**

The visual function of Iranian adults aged 45–69 years was moderate. The male gender, higher education and the higher economic status had a better visual function.

## Introduction

According to the WHO’s latest report, global estimates on visual impairment show that 2.2 billion people have a vision impairment and majority of them are over the age of 50 years [1]. According to the Global Vision Database Maps, about 217 million people have a moderate and severe visual impairment, of which 0.4% are blind; the global prevalence of distant visual impairment is also estimated to be 3.4%. According to the Lancet Global health 2021, more than 295 million people have a moderate and severe visual impairment, of which 0.55% are blind. Despite of a 27% reduction in proportion of blindness from 1990 up to now, the total number of people with blindness and moderate and severe vision impairment has increased significantly (by 51 and 92%, respectively) [2]. Chronic ocular diseases can significantly affect the quality of life of these patients, therefore, evaluating the visual quality can recognize the perception and feeling of visual performance and quality of life after ocular disease.

Vision-related quality of life (VRQoL) is an outstanding and measurable health outcome in patients with visual impairment [3], that indicates the impact of the chronic eye disease on daily activities [4]. The VRQoL can be evaluated by measuring the degree of disability experienced by a person in vision-related daily activities [5]. The 51-item National Eye Institute Visual Function Questionnaire (NEI-VFQ) was first designed in 1998 [6]. Then a shorter, 25 item, 12 subscales version, known as NEI-VFQ-25, was designed and standardized to give respondents more comfortable and improve data quality as well as to measure the health-related quality of life (HRQOL) [7]. The questionnaire has been translated and standardized in Italian, French, Spanish, German, Japanese [8], and also Persian [9].

The VRQoL in ocular disorders such as cataract [10], ocular graft-versus-host disease [11], conjunctivochalasis [12], optic neuropathy [13], and dry eye syndrome [14] have already been studied. Some eye surgeries such as vitrectomy can significantly improve the quality of life of patients with various vitreoretinal disorders [15]. A study of a Japanese population confirmed the negative association between VRQoL and the intensity of ocular diseases [12].

To the best of our knowledge, no population-based study has examined the VRQoL in Iran. Only a clinical-based study in 2012 on a clinical sample showed that the quality of life in patients with chronic ocular diseases was significantly reduced and was lower in comparison to patients in other countries [16].

This report aims to evaluate vision function and disability as well as related factors using the short-form visual functioning scale (SFVFS), which its validity and reliability have already been verified [9] in a population aged 45–69 years.

## Methods

### Participant and sampling

This study was conducted using data obtained from 4737 adults (aged 45–69 years) at the second phase of the Shahroud eye cohort study in 2014, aiming to identify the causes of eye diseases and visual impairment. The methodology and protocol of study have already been reported [17], but here is a summary.

In the first phase, in 2009, 5190 people aged 40–64 years were selected using random cluster sampling. In the second phase, in 2014, all participants were asked to participate in periodic examinations, where the recall welcomed by 4737 of them (the response rate: 91.3%). All participants underwent clinical and para-clinical examinations after conducting interviews.

### Short-form visual functioning scale (SFVFS)

SFVFS is derived from the NEI-VFQ-25 by Pesudovs et al. [18] They showed that NEI-VFQ is not unidimensional questionnaire and some items belong to socioeconomic construct. They segregated the visual functioning items into a scale and made a very effective measure of visual functioning [18]. The interviewer format SFVFS contains six questions; question 1 evaluates the vision of both eyes, and the results ranging from 1 (very good) to 6 (completely blind). Remaining questions which are rated with a 6-point Likert scale evaluate the level of the participant’s problems when performing vision-related activities (including text reading, job or leisure, finding things in box, reading driving sign and walking in low light), and the results ranging from 1 (no problem) to 6 (unable for other reasons).

In this study, disability was defined as limitation of activities related to visual problems.

### Statistical analysis

We used the Rasch model, which is a Random Coefficient Multinomial Logit Model, presented for the first time by Conrad et al. [19] for visual studies. Large-scale testing and construct validation research are main advantages of Rasch model. This is an Iterative approach that starts with the general fitting the data into the model and examining the threshold graph, which is used to determine the uneven thresholds. The main parameters in this analysis are thresholds rating and arrangement, fitness statistics and separation indices [20]. The Rasch Rating Scale (RRS) model is generally used for data with categorized rated responses. In this study, we used the modified Rasch scores. The scores range from -∞ to +∞, with the zero as the median score. Regarding the response variable values (1: no problem and 6: impossible/unfeasible), the more negative Rasch scores, the better the visual function and the lesser the visual disability.

Economic status was defined by the Principal Component Analysis on home assets [21]. Quantitative and qualitative data were described using mean ± standard deviation (SD) with 95% confidence interval (95% CI) and frequency (%), respectively. Multiple linear regression models were used to estimate the association between SFVFS (outcome index) and gender, education, marital status, insurance coverage and economic status (predictor variables).

### Ethical considerations

This study was conducted in accordance with the Helsinki Declaration. All procedures involving participants and related documentation, as well as the informed consent processes and form were approved by the Ethics Committee of Shahroud University of Medical Sciences (Reference number: 8737). We obtained written informed consent from all participants after explaining the methods and purpose of the research, allowing sufficient time for questions, and ensuring clarity. For subjects who were not able to read the consent materials, the approved oral process was to have the potential participants’ representative present the information verbally and serve as witness. If oral consent was granted, the fingerprint of the subject was captured, and the witness signed and dated the consent form. The member of the research team in charge of the consent process added a statement that the subject was unable to read the information and that an oral consent process was used.

## Results

Out of 4737 participants in this study, the visual function information of 4715 people aged 55.9 ± 6.2 years was available of which 1935 (58.5%) were male; 4221 (52.59%) of participants were married, 406 (8.61%) were widow and the rest were single and divorced;

The visual function of 75.3, 17.1 and 7.5% of participants were “very good or good”, “moderate”, and “bad or very bad”, respectively, while 0.06% were unable for vision. The running mean of visual function was calculated to be − 3.95 ± 0.02 in total sample.

The visual function status is presented in Table 1, in both visual activity-based and age group-based. When reading a text, the greatest visual problem was observed in the age group of 65–69 years old (9.5%) and 45–49 years old (9.2%), respectively. When finding things in a box, the greatest visual problem was observed at the age ranged 65–69 years old (2.8%) and 50–54 years old (1.5%), respectively. In the case of job or leisure, the greatest visual problem was observed in the group aged 45–49 years (18%).

**Table 1:** Visual functions and disability by age groups in adult population, Shahroud, Iran, 2014

Visual functions	Age group					Total
	45–49	50–54	55–59	60–64	65-69	
**Text Reading** N (%)						
No Problem	514 (58.9)	757 (59.5)	713 (60.4)	480 (55.8)	272 (51.0)	2736 (58.0)
Little Problem	173 (19.8)	257 (20.2)	208 (17.6)	145 (16.8)	93 (17.4)	876 (18.6)
Moderate Problem	70 (8.0)	79 (6.2)	73 (6.1)	42 (4.8)	27 (5.0)	291 (6.2)
High Problem	81 (9.2)	106 (8.3)	101 (8.5)	77 (8.9)	51 (9.5)	416 (8.8)
Unable for Vision	7 (0.8)	10 (0.7)	7 (0.5)	5 (0.5)	7 (1.3)	36 (0.8)
Unable for other reasons	27 (3.1)	62 (4.8)	78 (6.6)	110 (12.8)	83 (15.5)	360 (7.6)
**Job or Leisure** N (%)						
No Problem	447 (51.2)	665 (52.3)	634 (53.7)	438 (50.9)	270 (50.6)	2454 (52.0)
Little Problem	205 (23.5)	305 (24.0)	268 (22.7)	199 (23.1)	120 (22.5)	1097 (23.3)
Moderate Problem	41 (4.7)	86 (6.7)	71 (6.0)	53 (6.1)	34 (6.3)	285 (6.0)
High Problem	156 (17.8)	184 (14.4)	168 (14.2)	138 (16.0)	83 (15.5)	729 (15.5)
Unable for Vision	22 (2.5)	29 (2.2)	38 (3.2)	30 (3.4)	26 (4.8)	145 (3.1)
Unable for other reasons	1 (0.1)	2 (0.1)	1 (0.08)	1 (0.1)	0 (0.0)	5 (0.1)
**Finding Things in Box** N (%)						
No Problem	809 (92.7)	1138 (89.5)	1059 (89.7)	769 (89.5)	459 (86.1)	4234 (89.8)
Little Problem	41 (4.7)	89 (7.0)	79 (6.6)	52 (6.0)	40 (7.5)	301 (6.4)
Moderate Problem	8 (0.9)	17 (1.3)	17 (1.4)	18 (2.1)	13 (2.4)	73 (1.5)
High Problem	10 (1.1)	20 (1.5)	16 (1.3)	11 (1.2)	15 (2.8)	72 (1.5)
Unable for Vision	3 (0.3)	7 (0.5)	9 (0.7)	9 (1.0)	6 (1.1)	34 (0.7)
Unable for other reasons	1 (0.1)	0 (0.0)	0 (0.0)	0 (0.0)	0 (0.0)	1 (0.1)
**Reading Driving Sign** N (%)						
No Problem	724 (83.0)	1034 (81.3)	974 (82.5)	680 (79.1)	409 (76.7)	3821 (81.0)
Little Problem	86 (9.8)	130 (10.2)	117 (9.9)	94 (10.9)	63 (11.8)	490 (10.4)
Moderate Problem	40 (4.5)	68 (5.3)	48 (4.0)	48 (5.5)	26 (4.8)	230 (4.9)
High Problem	18 (2.0)	33 (2.6)	31 (2.6)	24 (2.7)	26 (4.8)	132 (2.8)
Unable for Vision	2 (0.2)	2 (0.1)	3 (0.2)	5 (0.5)	4 (0.7)	16 (3.4)
Unable for other reasons	2 (0.2)	4 (0.3)	7 (0.5)	8 (0.9)	5 (0.9)	26 (5.5)
**Walking in Low Light** N (%)						
No Problem	738 (84.6)	1088 (85.6)	982 (83.2)	701 (81.6)	421 (78.9)	3930 (83.3)
Little Problem	81 (9.2)	116 (9.1)	115 (9.7)	78 (9.0)	58 (10.8)	448 (9.5)
Moderate Problem	26 (2.9)	36 (2.8)	39 (3.3)	42 (4.8)	31 (5.8)	174 (3.7)
High Problem	23 (2.6)	23 (1.8)	39 (3.3)	32 (3.7)	18 (3.3)	135 (2.9)
Unable for Vision	3 (0.3)	6 (0.4)	4 (0.3)	6 (0.7)	5 (0.9)	24 (5.1)
Unable for other reasons	1 (0.1)	2 (0.1)	1 (0.08)	0 (0.0)	0 (0.0)	4 (0.5)

The visual function of the participants is presented in Table 2, in total, and in gender-based. In general, 58% of the participants did not have any problems when reading the text, while 8.8% of them experienced serious problems at the same conditions; 52% of people did not have any visual problems with performing a job or in their leisure, and 10% experienced inability (at various levels) in finding things in a box. When reading driving signs, 0.8% of participants did not saw any signs, and 2.8% had a severe problem in this regard; 83.3% of the participants were able to walk in low light.

**Table 2:** Visual functions and disability by gender in adult population, Shahroud, Iran, 2014

Visual functions	No Problem	Little Problem	Moderate Problem	High Problem	Unable for Vision	Unable for other reasons
**Text Reading** N (%)						
All	2736 (58.0)	876 (18.5)	291 (6.1)	416 (8.8)	36 (0.7)	360 (7.6)
Male	1237 (63.9)	327 (16.9)	113 (5.8)	158 (8.1)	8 (0.4)	92 (4.7)
Female	149 (53.9)	549 (19.7)	178 (6.4)	258 (9.2)	28 (1.0)	268 (9.6)
**Job or Leisure** N (%)						
All	2454 (52.0)	1,097 (23.2)	285 (6.0)	729 (15.4)	145 (3.0)	5 (0.1)
Male	1340 (48.2)	712 (25.6)	196 (7.0)	448 (16.1)	81 (2.9)	3 (0.1)
Female	1114 (57.5)	385 (19.9)	89 (4.6)	281 (14.5)	64 (3.3)	2 (0.1)
**Finding Things in Box** N (%)						
All	4234 (89.8)	301 (6.3)	73 (1.5)	72 (1.5)	34 (0.7)	1 (0.02)
Male	2461 (88.5)	203 (7.3)	44 (1.5)	47 (1.6)	25(0.9)	0 (0.0)
Female	1773 (91.6)	98 (5.0)	29 (1.5)	25 (1.2)	9 (0.4)	1 (0.05)
**Reading Driving Sign** N (%)						
All	3821 (81.0)	490 (10.3)	230 (4.8)	132 (2.8)	16 (0.3)	26 (0.5)
Male	2156 (77.5)	331 (11.9)	163 (5.8)	96 (3.4)	11 (0.4)	23 (0.8)
Female	1665 (86.0)	159 (8.2)	67 (3.4)	36 (1.8)	5 (0.2)	3 (0.1)
**Walking in Low Light** N (%)						
All	3930 (83.3)	448 (9.5)	174 (3.6)	135 (2.8)	24 (0.5)	4 (0.08)
Male	2229 (80.1)	300 (10.7)	124 (4.4)	106 (3.8)	18 (0.6)	3 (0.1)
Female	1701 (87.9)	148 (7.6)	50 (2.5)	29 (1.5)	6 (0.3)	1 (0.0)

The Rasch results are presented in Table 3. The results of the Multiple Linear Regression model showed that gender, education level, and economic status were factors affecting the visual function score. The running mean was lower in males (− 4.16) than females (− 3.81) (β = 0.14, 95%CI: 0.04–0.23; *p* = 0.005). The negative association between education and visual function indicated that visual function score decreases when the education level increases, leading the vision function to improve. For example, the visual function of people with academic education was more desirable than illiterate people (β = − 1.06, 95%CI: − 1.30; − 0.82; *p* < 0.001). Also, visual function in participants with moderate (β = 0.16, p = 0.005) and low (β = 0.28, p < 0.001) economic status was worse than those with high economic status. The relationship between age groups (*P* > 0.05), marital status (*P* = 0.370) and insurance coverage (*P* = 0.070) with visual function were not significant.

**Table 3:** Mean estimation and Multiple linear regression of visual functions by independent variables in Shahroud, Iran, 2014

Rmean		Mean	95% CI	Coefficient	95%CI	***p***-value
**Age group**	45–49	-3.98	-4.08, -3.86	Ref		
	50–54	-4.02	-4.11, -3.93	-0.08	-0.21, 0.05	0.26
	55–59	-4.00	-4.09, -3.90	-0.10	-0.24, 0.03	0.15
	60–64	-3.89	-4.01, -3.77	-0.10	-0.25, 0.05	0.19
	65-69	-3.74	-3.90, -3.59	-0.08	-0.26, 0.09	0.35
**Gender**	Male	-4.16	-4.23, -4.08	Ref (male=0, female=1)		0.005
	Female	-3.81	-3.88, -3.74	0.14	0.04, 0.23	
**Economic Status**	High	-4.26	-4.34, -4.17	Ref		
	Moderate	-3.94	-4.02, -3.86	0.16	0.04, 0.27	0.005
	Low	-3.64	-3.75, -3.54	0.28	0.14, 0.42	<0.001
**Education**	Illiterate	-3.15	-3.33, -2.97	Ref		
	Primary	-3.80	-3.90, -3.71	-0.59	-0.80, -0.39	<0.001
	Guidance	-3.96	-4.08, -3.84	-0.69	-0.90, -0.48	<0.001
	High School	-4.20	-4.28, -4.11	-0.88	-1.09, -0.67	<0.001
	College	-4.47	-4.59, -4.35	-1.06	-1.30, -0.82	<0.001

## Discussion

Based on the results, in adult population aged 45–69 years, the visual function of 75, 17.1 and 7.5% of participants were “very good and good”, “moderate”, and “bad and very bad”, respectively. About a quarter of the participants had visual function less than average. Most of the visual impairments were related to job or leisure, so that nearly half of participants had a weakness in this regard. The least visual disability when finding things in the box was in the near-vision sub-scale, and about 90% had no problem with their visual function. Similarly in Gall’s study, 177 patients (aged 21–83 years; with a history of stroke and visual field defect) had a mean score 65.25 for the near visual function, which was lower (worse) than the mean scores (72.75) for distance visual function [22]. The greatest visual problem in the age groups of 45–49 years and 65–69 years was observed when reading a text, while in the age group of 65–69 years; it was found when finding things in a box. Similar to us, a cross-sectional study on patients with diabetic retinopathy (aged 50–70 years) by Shrestha et al. [23] showed the greatest disability with near vision activity including legibility of sentences and letters and the least visual disability with clothing. The age was the similar role in current study and a study conducted by Shrestha et al. [23], where the majority of participants were in the age group of over 50 years old and reading or writing a text was the most common visual disability observed.

In contrast to present results, a population-based study [24] examined the visual function in 12,231 German participants with mean age 66.95 ± 14.7 years and showed a significant correlation between the lower scores and higher ages (− 0.48, *P* < 0.001). The different age groups and age range of participants, sample size, life style (in the term of hours spending for reading, hubbies, work and …), education level, culture (living alone or with family) and accessibility to medical and supportive services can justify the differences in the results of the two studies.

The present study showed that the visual function in females was worse than that of males. Gall et al. reported that the visual function score of females was 1.57 less than males [22], under constant age, socioeconomic status and visual acuity [24]. As shown in previous studies [25, 26], females receive less eye care services for vision correction. Also, the complications of cataract surgery in females are greater than males while they receive less postoperative cares and services [27]. Also, gender differences can be related to fewer problems in social functioning in females [28].

There was a positive correlation between visual function with increasing education level and improving the economic status in the current study. Similarly Ulldemolins et al. [29] showed a negative correlation between the improved socioeconomic status (e.g., higher education, high income, non-manual occupational social class, etc.) with the prevalence of blindness or visual impairment in a review study.

World Health Organization recommend a framework known as International Classification of Functioning, Disability and Health (ICF) for classification of health and health-related domains since 2001 [30]. To the best of our knowledge there is no report, comparing SFVFS with ICF or even the new WHODAS 2.0 for measuring health and disability. The comparison of these tools needs new and comprehensive studies.

Standard design, large sample size, population-based design and response rate of over 90% are among the strengths of this study. The impossibility of generalizing the results to the entire population of Iran may be considered as a limitation of this study. It should be also noted that visual function associated factors, investigated in regression model, do not necessarily have a causal effect in this cross-sectional study.

## Conclusion

Only 7.5% of adults aged 45–69 years old had bad or very bad visual function. However, it was more impaired in females, low educated and low economic groups which should be considered for policymaking.

## Data Availability

The datasets used and/or analyzed during the current study are available from the corresponding author on reasonable request.

## Abbreviations

SFVFS: Short-Form Visual Functioning Scale
NEI-VFQ: National Eye Institute Visual Functioning Questionnaire
VRQoL: Vision-related quality of life
CI: Confidence Intervals
SD: Standard Deviation
